# Straw in Clay Bricks and Plasters—Can We Use Its Molecular Decay for Dating Purposes?

**DOI:** 10.3390/molecules25061419

**Published:** 2020-03-20

**Authors:** Johannes Tintner, Kimberly Roth, Franz Ottner, Zuzana Syrová-Anýžová, Ivana Žabičková, Karin Wriessnig, Roland Meingast, Hubert Feiglstorfer

**Affiliations:** 1 Institute of Physics and Materials Science, University of Natural Resources and Life Sciences, Peter Jordan Straße 82, 1190 Vienna, Austria; kim.roth@hotmail.com; 2 Institute Applied Geology, University of Natural Resources and Life Sciences, Peter Jordan Straße 82, 1190 Vienna, Austria; franz.ottner@boku.ac.at (F.O.); karin.wriessnig@boku.ac.at (K.W.); 3 National Heritage Institute, Valdštejnské náměstí 162/3, 1118 01 Praha, Czech Republic; syrova@sovamm.cz; 4 Faculty of Architecture, University of Technology, Poříčí 273/5, 639 00 Brno, Czech Republic; zabickova@gmail.com; 5 Lopas GmbH, Oberwaltersdorfer Straße 2c, 2523 Tattendorf, Austria; roland.meingast@lopas.at; 6 Institute for Social Anthropology, Austrian Academy of Sciences, Hollandstraße 11–13, 1020 Vienna, Austria; hubert.feiglstorfer@oeaw.ac.at

**Keywords:** adobe construction, earth construction, vernacular architecture, straw amendments, FTIR spectroscopy

## Abstract

Dating of clay bricks (adobe) and plasters is a relevant topic not only for building historians in the Pannonian region. Especially in vernacular architecture in this region, clay with straw amendments is a dominant construction material. The paper presents the potential of the molecular decay of these amendments to establish prediction tools for age based on infrared spectroscopic measurements. Preliminary results revealed spectral differences between the different plant parts, especially culms, nodes, and ear spindles. Based on these results, a first prediction model is presented including 14 historic samples. The coefficient of determination for the validation reached 62.2%, the (RMSE) root mean squared error amounted to 93 years. Taking the limited sample amount and the high material heterogeneity into account, this result can be seen as a promising output. Accordingly, sample size should be increased to a minimum of 100 objects and separate models for the different plant parts should be established.

## 1. Introduction

Clay is one of the oldest construction materials in mankind. Various building techniques have been developed according to locally available raw material and building traditions. Results of building techniques are, for example, rammed earth walls, cob walls, clay bricks, clay plasters, etc. Terms used for the clay itself, such as earth, loam, mud, etc., may vary regionally. Especially in dry regions, remains of clay constructions are among the oldest testimonies of human settlements, whether in the Andes, Central Asia, the dry area of settlement around the Sahara Desert from Mali to Ethiopia and Egypt, or even the prolific region from the Levante to the delta of Euphrat and Tigris [1]. In comparison to other construction materials like wood or stone, scientific research on these constructions is rather scarce. In Austria, the assessment of traditional knowledge and the experimental proof of old techniques has become a rising field of interest in recent years [2]. For some years, the use of clay has faced certain renaissance since building physical properties that account for indoor climate have become increasingly popular. A striking aspect for this development is the low carbon footprint of the material in comparison to reinforced concrete or metal [3]. 

Straw is used in clay constructions for different purposes, mainly to reduce weight and as reinforcement [4]. A reduction of shrinkage of the clay when using straw is a method in archaeology commonly mentioned as tempering. Chemically, straw consists mainly of lignocellulosic complexes and mineral compounds [5]. Dating of buildings can be done using different methods. Among natural scientific methods, dendrochronology and radiocarbon dating are most common. Besides their massive strength, both methods incorporate intrinsic drawbacks resulting in objects that cannot be dated. Important drawbacks for clay constructions are the lack of sufficient amount of construction wood or tree ring patterns that do not fit to any reference curve [6,7]. Molecular changes of organic materials with comparable chemical structure have been documented [8,9]. The idea to use molecular changes for the discrimination of different ages has also been discussed for charcoal [10], since wood-dating tools based on the molecular decay already have been developed [11]. These models gave reasons for the presented study, as the molecular composition of straw and wood contains the same main components [12]. Infrared spectroscopy is a suitable method to describe molecular changes. Cheap and rapid handling allows a huge amount of measurements. Therefore, even huge sample sets can be assessed with a considerable number of replicates covering material heterogeneity. This is a standard method for investigations of aging processes of organic matter in the environment [13].

The objective of this work was to prove whether the molecular decay of straw in clay bricks and plasters measured by means of (FTIR) Fourier Transform Infrared spectroscopy can be used for dating purposes. As a precursor of this proof, we had to check the homogeneity of different straw parts in their spectral fingerprint.

## 2. Results

### 2.1. Spectral Pattern of Different Straw Parts

**Figure 1:**
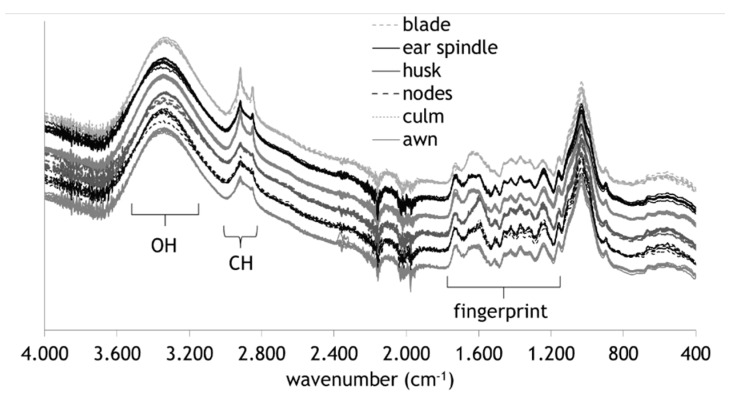
Infrared spectra of different plant parts of wheat straw (8 repl. each). Spectra are shifted on the vertical axis; legend sequence corresponds to the shifted spectra sequence. Broad bands of OH and CH functional groups are indicated, as well as the fingerprint region.

First, the results of the preparatory study are presented. FTIR spectra of the different plant parts showed systematic differences in different spectral regions (Figure 1) (For more please see Supplementary Materials). Husks, ear spindles, and blades have significantly sharper band maxima in the region of CH stretching vibrations between 3000 and 2800 cm^−1^ originating mainly from methyl and methylene groups [14]. Nodes and, to a smaller degree, culms have a comparably sharp band in the fingerprint region ranging from 1580 to 1600 cm^−1^. This could be assigned to asymmetric stretch vibration of carboxylates [15,16]. Even more likely, it could arise from the higher amount of lignin in nodes [17]. The band can also be assigned to the aromatic skeletal ring vibration predominantly of syringyl alcohols [14], a typical major lignin monomer in grass lignin [18]. 

**Figure 2:**
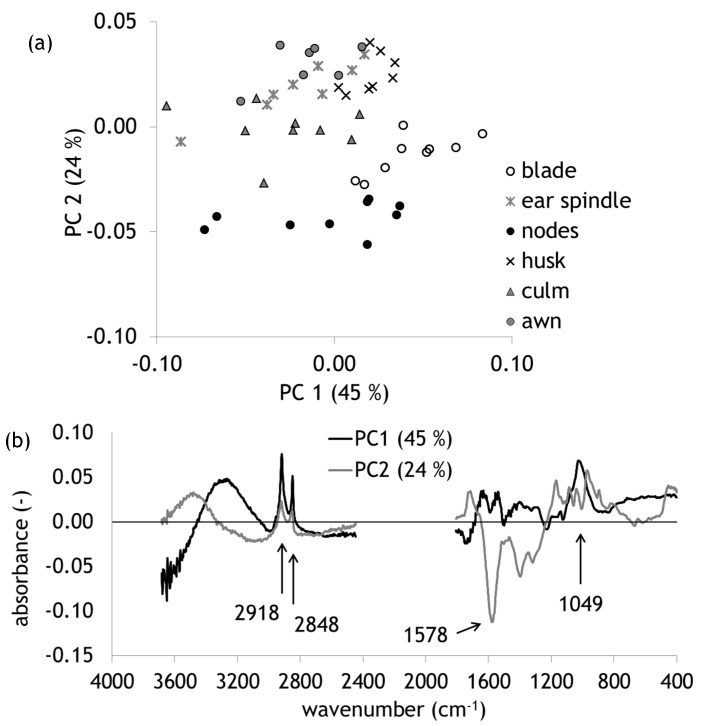
Principal Component Analyses (PCAs) of different plant parts of wheat straw n = 51; (**a**) scores plot and (**b**) loadings plot of the first two PCs, maxima, and minima discussed in the text are marked with an error and positions are given.

To evaluate even smaller differences Principal Component Analysis (PCA) has been performed; the results are presented in Figure 2. Figure 2a displays in the scores plot the different plant parts arranged according to their chemical fingerprints. In Principal Component (PC) 1 especially blades are shifted to the right. PC 2 separates the nodes to the bottom and husks, ear spindles, and awns to the upper part. Figure 2b presents the loadings plots that explain the pattern visible in the scores plot. PC 1 is dominated by the methyl and methylene bands (2918 and 2848 cm^−1^) as already visible in Figure 1. Additionally, a maximum at 1049 cm^−1^ is visible that can be assigned to the valence vibration of C-O mainly from C3-O3H [19] found especially in cellulose. PC 2 is dominated by a minimum at 1578 cm^−1^ assigned to the aromatic ring of lignin [14]. Variability within the different plant parts was found to be smaller than between them.

As differences in the spectral pattern became evident, the different plant parts were separated in the historic samples. 

### 2.2. Mineral Matrix of Historical Samples

The description of the preservation conditions is based on the examination of the mineral compounds of plaster and adobe material (Table 1). The mineral composition represents the expectable material in the region of Bohemia and the Viennese Basin including Moravia and Lower Austria [20]. In all samples, quartz can be found in high amounts or even dominant. Additionally, mica and feldspars are found in low or high amounts. The Viennese Basin contains considerable amounts of calcite and dolomite mirrored in all corresponding samples. Clay minerals are found in all samples, but only in the samples from Moravia and Lower Austria in low to high amounts. Swellable clay minerals are found only in the samples from Lysovice and Kučerov.

### 2.3. Molecular Changes over Time

**Figure 3:**
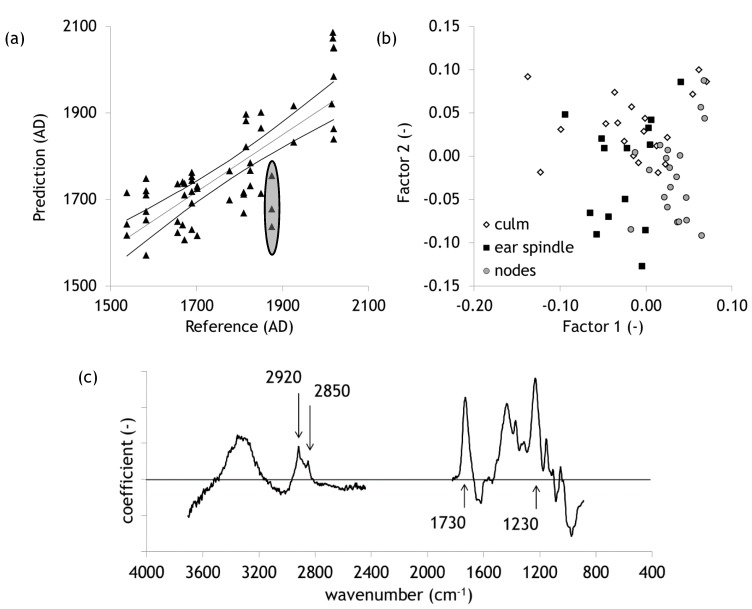
Results of Partial Least Squares (PLS) regression n = 53. (**a**) Predicted vs. reference age, perfect fit, and confidence bands are given *α* = 0.05, (**b**) scores plot of the first two factors of the PLS-model, (**c**) loadings plot for the regression coefficient of the model.

Due to the limited number of objects spectra of all plant parts were combined in a data set for a preparatory model to predict the age of a sample out of its spectral pattern. Results of the Partial Least Squares (PLS) regression are found in Figure 3. The model establishes a coefficient of determination for the validation of 62.2%. The root mean squared error amounts to 93 years. This value refers to a single sample. For practical purposes, an object could be samples with replicates as well. These replicates will narrow the prediction interval of the estimation of object’s age. Figure 3b displays the scores of the first two factors. It becomes obvious that the different plant parts behave slightly different. This variability is part of the overall model variability. The loadings plot in Figure 3c shows which spectral regions are most relevant for the model prediction. They indicate which molecular groups dominate the aging effects that are used for our model. Dominant maxima are found at the 1730 cm^−1^ and 1230 cm^−1^. These two bands already have been found to be the most important ones in the taphonomy of wood and bark in a prehistoric salt mine in Hallstatt, Upper Austria. They were assigned to acetyl groups of hemicelluloses [8,9]. Acetyl groups can be seen as one of the weakest parts of the molecules. For wood, the deterioration of this molecular group during aging has been observed a number of times [8,11,13,21,22]. Methylene groups (band maxima 2920 and 2850 cm^−1^) play only a subordinate role in the molecular changes over time. This group is commonly affected by biological degradation of organic matter [23].

## 3. Discussion

The results indicate several important tasks that have to be taken into consideration, when planning a robust and widely applicable prediction model for age based on molecular decay. The study of different parts of straw demonstrated significant differences in the chemical fingerprint of these materials. These results coincide with literature that proved different ratios of the main compounds of the lignocellulosic complex in the different plant parts [17]. It must be assumed that in proceeding works, the different plant parts will be segregated into separate models.

Besides several other potential flaws, it must be stressed that the dating of several objects included in the data set is rather uncertain. Looking at Figure 3a, the data points of the object Kučerov, Moravia–1875 are rather different from what we would expect them according to spectral characteristics (marked in the plot by an ellipse). Withdrawing them and recalculating the model leads to a RMSE of 85 years and r^2^ of 67%. Even if there were no evidence, there would be no point in taking the object out of the data set (we must keep in mind that the low amount of samples increases the susceptibility of incorrect dates in our Y-matrix). Potential reasons could be an incorrect description of building history or just a recycling of material from an older object. Covering the whole range of variability can only be achieved by a substantial increase of objects. Thereby, the risk of doubtful dating results is spread over a wider range. We propose a minimum of 100 objects to be included in a model that can be practically applied.

Another influence factor that should be taken into consideration is the inorganic matter of the constructions. Clay minerals are known to preserve organic material well. Besides clay plasters, we can expect straw also in lime plasters, too. The high pH above 13 might result in different molecular changes leading to separate models. The current heterogeneity of mineral compounds visible in the results of mineral analyses is rather low, but an impact cannot be excluded without fail at the current stage. Far more samples with different mineral compositions of the surrounding clay are necessary to investigate dependencies of aging effects. We expect that these effects play a minor role and therefore will get lost in the overall prediction error. Clay minerals especially have strong impact on microbial activity [24,25]. This is an important issue for the straw to outlast embedded in clay architecture. We guess that clay constructions contain an excessive supply of clay to reduce microbial activity strongly. An analytical proof will be an important task for the future.

We conclude that the presented model is a promising first result. It demonstrates the high potential of molecular decay of straw amendments for dating purposes. 

## 4. Materials and Methods

### 4.1. Materials

For a preparatory study, recent wheat straw was sampled and different parts of the above ground biomass were separated: culm, blade, husk, nodes, ear spindle, and awn. Eight replicates of each plant part were measured.

Historical buildings were sampled in the Czech Republic (reference material and recent constructions originated from Austria (Table 2). Brick and plaster samples originated from different objects. An object refers to a certain construction phase of a building. In two buildings, two construction phases were sampled (Kučerov and one building in Čistá), and in Vračovice three were sampled. Samples from Niedersulz in Lower Austria were used as reference material and originated from walls constructed in the course of a student’s university program made from clay bricks. Additionally, the samples from Lysovice and Kučerov were taken from adobe bricks, whereas all other samples originated from clay plasters. Sample “Vračovice, 1926” has been a rush mat. Dating of Bohemian objects has been performed by means of dendrochronology and has already been published [26,27]. Moravian objects were chronologically described based on historical information. This concerns objects with a maximum age of approximately 200 years. Recent reference wheat straw has been taken from a horse stable. Reed reference has also been included in the data set.

All samples were taken from the inner parts of the material, in minimum 0.5 cm of coverage protected them against ambient air. This distance was found to be critical for wood [11].

### 4.2. Methods

#### 4.2.1. Sample Preparation

For the preparatory study, the different parts of wheat straw were separated, water-washed in an ultrasonic bath, dried at 105 °C and milled with an ultracentrifugal mill to a particle size below 20 µm. According to the results of this preparatory study, culms, nodes, and ear spindles were separated as they were found in most of the samples (blades, husks, and awns were either not found or only in negligible quantity in most of the samples). The gentler vibratory disk mill was used instead of the ultracentrifugal mill (which reaches higher temperatures). Milling time was set in correspondence with the sample amount, ranging from 120 s for around 600 mg to 30 s for around 50 mg.

Around 5 g of inorganic compounds of the bricks and plasters were milled by hand with mortar and pestle.

#### 4.2.2. Fourier Transform Infrared (FTIR) Spectroscopy and Statistical Evaluation

FTIR spectra were recorded in the Attenuated Total Reflection (ATR) mode in the mid infrared area (4000–400 cm^−1^) with an optical crystal of a Bruker^®^ Helios FTIR micro sampler (Tensor 27). This device allows spot measurements with a spatial resolution of 250 micrometer. 32 scans were recorded at a spectral resolution of 4 cm^−1^. Spectra were vector normalized using the OPUS ^©^ (version 7.2) software. For the preparatory study, eight replicates per plant part were taken, and for the main sample set five replicates per sample were averaged. Principal Component Analysis (PCA) and Partial Least Squares (PLS) regression have been performed using The Unscrambler X 10.1 (© Camo). The regression model was performed with five factors. These factors include the information of the spectral range, but with different loadings. Therefore, the different factors can deliver different chemical information. All factors are combined in the final model. The PLS algorithm decides based on data how many factors should be included. The spectral range focused on the areas with dominating bands of straw organic matter including 3700 to 2440 cm^−1^ and 1820 to 890 cm^−1^. Any influence of remaining inorganic compounds is therefore limited. The model has been 10-fold cross-validated.

#### 4.2.3. X-ray Diffractometry

The assessment of mineral compounds has been performed by means of X-ray diffractometry (XRD). A Panalytical^®^ X’Pert Pro MPD diffractometer was used with automatic divergent slit, Cu LFF tube (45 kV, 40 mA), with an X’Celerator detector. The measuring time was 25 s, with a stepsize of 0.017°. Diffractograms were recorded from 5° to 70° (2θ). Semiquantitative mineral composition of the bulk samples was estimated using Rietveld refinement with the Panalytical software © X’Pert HighScore Plus.

## Figures and Tables

**Table 1:** Results of (XRD) X-ray diffractometry, quar: quartz, calc: calcite, dolo: dolomite, fsp: feldspar, sm-v: swellable clay minerals: smectite and vermiculite, kaol: kaolinite, chlo: chlorite, amp: amphibole, gyps: gypsum; *** dominant, ** high amount, * low amount, traces.

Village, Year (AD)	Quar	Calc	Dolo	Mica	Fsp	Sm–V	Kaol	Chlo	Amp	Gyps
Niedersulz, 2017	**	**	**	**	**			**	*	
Niedersulz, 2015	**	**	**	**	**			**	*	
Kučerov, ~1875	***	*	.	*	*	*			.	
Lysovice, ~1850	**	**	*	*	*	**	*		.	
Kučerov, ~1825	**	*	.	*	*	*	*	*	.	
Zvonovice, ~1815	**	**	*	**	*			*		.
Benátky, 1810	**			*	*		.			
Vračovice, 1777	**			*	*		*		.	
Čistá, 1702	**			*	*		.		.	
Čistá, 1689	**			*	*		.			
Čistá, 1672	**			*	*		.			
Benátky, 1668	**	*		**	**		*	*	.	
Vraclav, 1651	***			.	*		*			
Čistá, 1583	**			*	*		.			
Vračovice, 1538	***			*	*		*		.	

**Table 2:** Sample description for the prediction model, location of the buildings and construction years are given, NO = nodes, EA = ear spindle, CU = culm.

Location	Year (AD)	Plant Parts
Recent reference material	2019	NO, EA, CU
Niedersulz, Lower Austria	2017	NO, CU
Niedersulz, Lower Austria	2015	NO
Vračovice, Bohemia *	1926	NO, CU
Kučerov, Moravia	~1875	NO, EA, CU
Lysovice, Moravia	~1850	NO, EA, CU
Kučerov, Moravia	~1825	NO, EA, CU
Zvonovice, Moravia	~1815	NO, EA, CU
Benátky u Litomyšle, Bohemia **	1810	NO, EA, CU
Vračovice, Bohemia **	1777	NO, CU
Čistá u Litomyšle, Bohemia **	1702	NO, EA, CU
Čistá u Litomyšle, Bohemia **	1689	NO, EA, CU
Čistá u Litomyšle, Bohemia *	1672	NO, EA, CU
Benátky u Litomyšle, Bohemia *	1669	NO, CU
Vraclav, Bohemia *	1651	NO, EA, CU
Čistá u Litomyšle, Bohemia *	1583	NO, EA, CU
Vračovice, Bohemia *	1538	NO, EA, CU

* [26]; ** [27].

## Supplementary Materials

The following are available online at https://www.mdpi.com/1420-3049/25/6/1419/s1. Infrared spectral data are made available as supplementary material.

## References

[1] Schroeder H., Feiglstorfer H. (2016). Building with earth—The current situation of a traditional construction. Earth Construction & Tradition.

[2] Meingast R., Feiglstorfer H., Feiglstorfer H. (2018). Earth building history in eastern Austria. Earth Construction & Tradition.

[3] Pacheco-Torgal F., Jalali S. (2012). Earth construction: Lessons from the past for future eco-efficient construction. Constr. Build. Mater..

[4] Laborel-Préneron A., Aubert J., Magniont C., Tribout C., Bertron A. (2016). Plant aggregates and fibers in earth construction materials: A review. Constr. Build. Mater..

[5] Hurter A.M. Utilization of annual plants and agricultural residues for the production of pulp and paper. Proceedings of the Pulping Conference.

[6] Speer J.H. (2010). Fundamentals of Tree-Ring Research.

[7] Stokes M.A., Smiley T.L. (2008). An Introduction to Tree-Ring Dating, [Nachdr.].

[8] Tintner J., Smidt E., Tieben J., Reschreiter H., Kowarik K., Grabner M. (2016). Aging of wood under long-term storage in a salt environment. Wood Sci. Technol..

[9] Tintner J., Smidt E., Aumuller C., Martin P., Ottner F., Wriessnig K., Reschreiter H. (2018). Taphonomy of prehistoric bark in a salt environment at the archaeological site in Hallstatt, Upper Austria—An analytical approach based on FTIR spectroscopy. Vib. Spectrosc..

[10] Smidt E., Tintner J., Klemm S., Scholz U. (2017). FT-IR spectral and thermal characterization of ancient charcoals—A tool to support archeological and historical data interpretation. Quat. Int..

[11] Tintner J., Spangl B., Reiter F., Smidt E., Grabner M. (2020). Infrared spectral characterization of the molecular wood decay in terms of age. Wood Sci. Technol..

[12] Williams C.L., Emerson R., Tumuluru J.S. (2017). Biomass Compositional Analysis for Conversion to Renewable Fuels and Chemicals. Biomass Volume Estimation and Valorization for Energy.

[13] Smidt E., Schwanninger M., Tintner J., Bohm K., Freitas L. (2013). Ageing and Deterioration of Materials in the Environment – Application of Multivariate Data Analysis. Multivariate Analysis in Management, Engineering and the Sciences.

[14] Schwanninger M., Rodrigues J., Pereira H., Hinterstoisser B. (2004). Effects of short-time vibratory ball milling on the shape of FT-IR spectra of wood and cellulose. Vib. Spectrosc..

[15] Nopp-Mayr U., Zohmann-Neuberger M., Tintner J., Kriechbaum M., Rosenberger R., Nopp H., Bosa A., Smidt E. (2019). From plants to feces: pilot applications of FTIR spectroscopy for studies on the foraging ecology of an avian herbivore. J. Ornithol..

[16] Smith B.C. (1999). Infrared Spectral Interpretation. A Systematic Approach.

[17] Ghaffar S.H., Fan M. (2013). Structural analysis for lignin characteristics in biomass straw. Biomass Bioenergy.

[18] Del Río J.C., Lino A.G., Colodette J.L., Lima C.F., Gutiérrez A., Martinez M.J., Lu F., Ralph J., Rencoret J. (2015). Differences in the chemical structure of the lignins from sugarcane bagasse and straw. Biomass Bioenergy.

[19] Marechal Y., Chanzy H. (2000). The hydrogen bond network in I β cellulose as observed by infrared spectrometry. J. Mol. Struct..

[20] Tollmann A. (1985). Geologie von Österreich.

[21] Pizzo B., Pecoraro E., Alves A., Macchioni N., Rodrigues J. (2015). Quantitative evaluation by attenuated total reflectance infrared (ATR-FTIR) spectroscopy of the chemical composition of decayed wood preserved in waterlogged conditions. Talanta.

[22] Pedersen N.B. (2015). Microscopic and Spectroscopic Characterisation of Waterlogged Archaeological Softwood from Anoxic Environments. Ph.D. Thesis.

[23] Smidt E., Eckhardt K.-U., Lechner P., Schulten H.-R., Leinweber P. (2005). Characterization of different decomposition stages of biowaste using FT-IR spectroscopy and pyrolysis-field ionization mass spectrometry. Biodgegradationchem.

[24] Williams L.B., Holland M., Eberl D.D., Brunet T., DeCourrsou L.B. (2004). Killer clays! Natural antibacterial clay minerals. Mineral. Soc. Bull..

[25] Dastjerdi R., Montazer M. (2010). A review on the application of inorganic nano-structured materials in the modification of textiles: Focus on anti-microbial properties. Colloids Surf. B: Biointerfaces.

[26] Syrová Z., Syrový J. (2013). Historic daubed corner-timbered constructions in Czech Republic. Proceedings of the Vernacular Heritage and Earthen Architecture.

[27] Škabrada J., Syrová-Anýžová Z. (2018). Nejstarší venkovské domy ve východních Čechách.

